# EZH2 inhibitors-mediated epigenetic reactivation of FOSB inhibits triple-negative breast cancer progress

**DOI:** 10.1186/s12935-020-01260-5

**Published:** 2020-05-19

**Authors:** Ruishan Zhang, Xiang Li, Zhuangkai Liu, Yuying Wang, Hao Zhang, Hong Xu

**Affiliations:** Department of Breast Cancer, Cancer Hospital of China Medical University, Liaoning Cancer Hospital and Institute, 44 Xiaoheyan Road, Dadong District, Shenyang, 110042 Liaoning People’s Republic of China

**Keywords:** TNBC, EZH2, FOSB, C/EBPβ, Epigenetic inactivation

## Abstract

**Background:**

Triple-negative breast cancer (TNBC) is the most aggressive subtype of breast cancer that lacks expression of estrogen receptor (ER) and progesterone receptor (PR) and the human epidermal growth factor receptor 2 (HER2) gene. Chemotherapy remains the standard of care for TNBC treatment, but considerable patients are very resistant to chemotherapy. Mutations or aberrant upregulation of EZH2 occur frequently, and EZH2 inhibitor (EZH2i) showed some preclinic antitumor effects in TNBC.

**Methods:**

RNA-seq data of 3 TNBC cell lines either treated with 2 μM GSK343, or stably transduced with shEHZ2, compared to untreated controls (GSE112378) were analyzed by Limma R package. The Kaplan–Meier plotter (KM plotter) database was used to assess the relevance of FOSB mRNA expression to relapse-free survival (RFS) in TNBC. Cell number counting and colony formation assays were used to detect the biological effect of FOSB on the growth of TNBC cells in vitro. The effect of FOSB on TNBC tumor growth in vivo was investigated in a mice tumor xenograft model. Luciferase reporter and chromatin immunoprecipitation (Chip) assays were used to determine the regulatory roles of C/EBPβ on FOSB expression.

**Results:**

We found that FOSB, a member of the activator protein-1 complex, was a direct downstream target of EZH2. FOSB was significantly decreased in TNBC samples and associated with better relapse-free survival (RFS). EZH2-mediated histone 3 trimethylated on lysine 27 (H3K27me3), a marker of silent chromatin conformation, at the FOSB promoter inhibited it expression. Depletion of FOSB in TNBC cells promoted cell proliferation in vitro and tumor growth in vitro by inactivating the p53 pathway and conferred resistant to EZH2 inhibitor (EZH2i). Mechanistically, EZH2i promotes the shift from H3K27me3 to H3K27ac at the FOSB promoter, and recruits the transcription factor C/EBPβ to activate FOSB gene transcription.

**Conclusion:**

Together, our results suggest that EZH2-mediated epigenetic inactivation of FOSB promotes TNBC expression and demonstrate that reactivation of FOSB expression by C/EBPβ underlies the anti-TNBC action of EZH2is.

## Background

Breast cancer is a heterogeneous disease that differs in morphology, molecular biology, clinical manifestations, and responsiveness to treatment [1]. In 2016, approximately 300,000 women were diagnosed with breast cancer and ~ 30,000 died (American Cancer Society: Breast cancer facts and figures 2015–2016). Based on the molecular phenotype of breast cancer, it is generally divided into four subtypes: the mammary glandular type, the human epidermal growth factor-2 overexpression type, the normal cell-like type, and the basal-like type [2]. Triple-negative breast cancer (TNBC) is an aggressive basal-like subtype that constitutes 12–18% of breast cancer patients and frequently develops resistance to chemotherapy [3]. TNBC patients lack the estrogen receptor (ER), progesterone receptor (PR) and HER2 receptor and therefore are not eligible for hormone or anti-Her2 therapy. The lack of targeted therapies and the poor prognosis of patients with TNBC warrants novel molecular targets to treat these patients [4]. Different strategies were used to treatment breast cancer in preclinical models [5–7]. In recent years, epigenetic mechanisms have been discovered to play an important role in the development of TNBC and targeting epigenetic enzymes might represent a novel treatment for TNBC patient [8, 9].

EZH2 is the enzymatically active core subunit of the PRC2 complex, which contains EED, SUZ12, and RbAp46/48. PRC2 methylates the lysine residue at position 27 of histone 3 (H3K27me3) to facilitate chromatin compaction and gene silencing [10, 11]. EZH2 was firstly identified as an oncogene in prostate and breast cancer, which is highly expressed in hormone-resistant and metastatic prostate cancers and TNBC with lower overall survival [12, 13]. Subsequent research found that EZH2 was highly expressed in a variety of malignancies, including head and neck cancer, bladder cancer, colorectal cancer, and non-small cell lung cancer, and was associated with poor prognosis [10]. High levels of EZH2 were shown to correlate with aggressiveness and advanced disease in each of these cancer types [10]. EZH2 has been shown to be essential for proliferation of cancer cell lines that is largely dependent upon its methyltransferase domain [14]. Forced expression of EZH2 leads to neoplastic transformation of breast epithelial cells and development of myeloproliferative disorder in mice [15]. Recurrent heterozygous point mutations at tyrosine 641 (Y641) within the C-terminal catalytic SET domain of EZH2 occur in 22% of germinal center B-cell (GCB) diffuse large cell B-cell lymphomas (DLBCL) and in 7% to 12% of follicular lymphomas (FL) [16, 17]. The mutant EZH2 caused aberrant H3K27me3 that leaded to repression of Polycomb target genes. Given the evidence for EZH2 enzymatic gain-of-function being a cancer driver, development of specific EZH2 inhibitors (EZH2is) has received widespread attention [18]. The existing preclinical research evidences show that EZH2is only have a certain therapeutic effect on individual hematological tumors containing EZH2 activating mutations [19]. For example, two EZH2 inhibitors, GSK343 and EPZ-6438 demonstrated preliminary benefits in DLBCL and FL [20, 21]. However, EZH2is are basically ineffective in the treatment of solid tumors, which greatly limits the clinical application of these inhibitors. A recent study found that EZH2is can inhibit H3K27me3 while promoting H3K27ac, resulting in epigenetic reactivation of oncogenic genes transcription [22]. Therefore, the identification of the target genes and downstream signals that were abnormally activated after EZH2is treatment might be beneficial for clarifying the causes of EZH2is resistance in solid tumor cells.

AP-1 transcription factor consists of a variety of dimers from the members of the JUN proto-oncogene (c-JUN) family (c-JUN, JUNB, JUND) or FBJ murine osteosarcoma viral oncogene homolog (FOS) family (c-FOS, FOSB, FRA1/2) [23]. AP-1 has been implicated in lots of biological processes including cell proliferation, death, differentiation and oncogenic transformation [24, 25]. For example, c-FOS could promote the cancer stem-like cell properties in head and neck squamous cell carcinoma [26]. However, the role of FOSB in TNBC has not been fully revealed. In this study, we identified FOSB as a novel EZH2 downstream target gene in TNBC, and inhibition of which resulted in tumor growth and EZH2is resistance.

## Materials and methods

### Clinical samples and data acquisition

Transcriptome RNA-sequencing (RNA-seq, FPKM) data of TNBC were downloaded from the TCGA data portal (https://cancergenome.nih.gov/), which contained data from 115 primary TNBC and 113 non-tumor tissues. RNA-seq data (RPKM) of 3 TNBC cell lines either treated with 2 μM GSK343, or stably transduced with shEHZ2, compared to untreated controls (GSE112378) were used for analysis by the R software Linear Models for Microarray and RNA-Seq Data (Limma) package (http://bioconductor.org/packages/Limma/). We performed differential gene analysis of all transcriptional data, setting a log2 |fold change| > 1 and a false discovery rate (FDR) < 0.05 as the cutoff values. The Wilcox-test was used for analyses. In 115 cases of TNBC RNA-Seq data (FPKM), FOSB was analyzed by GSEA 4.0.1 (with h.all.v7.0.symbols.gmt as the background gene set).

### Cell lines and reagents

Breast cancer cell lines MDA-MB-231 and MDA-MB-436 cells were purchased from Cell lines Cell Bank of Chinese Academy of Sciences; HEK293T cells were kept in our lab for routine work.

Cells were cultured in Dulbecco’s modified Eagle Medium (DMEM; Gibco BRL, Grand Island, NY, USA) with 10% FBS (Gibco, Gaithersburg, MD, USA), 100 U/ml penicillin and 100 μg/ml streptomycin, and maintained at 37 °C in a humidified chamber (5% CO_2_). All cell lines were routinely tested negative for Mycoplasma. GSK343 was purchased from Medchemexpress (HY-13500). Cycloheximide (239764) was purchased from Calbiochem (San Diego, CA, USA). The proteasome inhibitor MG132 was purchased from Sigma-Aldrich (St. Louis, MO, USA).

### RNA isolation, siRNAs and real-time PCR

Total RNAs from cells were extracted by using Trizol reagents (Invitrogen, Shanghai). The mRNAs were then reversed transcribed into complementary DNA (cDNA) using the Promega Reverse Transcription System (Madison, WI, USA). Oligo dT was used to prime cDNA synthesis. Real-time PCR was performed using a SYBR Green Premix Ex Taq (Takara, Japan) on a Light Cycler 480 (Roche, Switzerland).The mRNA levels of GAPDH were used as internal control. Differences in gene expression were calculated using the 2−ΔΔCt method and expressed as fold-changes. PCR conditions included an initial holding period at 95 °C for 5 min, followed by a two-step PCR program consisting of 95 °C for 5 s and 60 °C for 30 s for 50 cycles. Primers used for qPCR analysis were list as follows: FOSB forward, 5′-GCTGCAAGATCCCCTACGAAG-3′; reverse, 5′-ACGAAGAAGTGTACGAAGGGTT3′; EZH2 forward, 5′-ATCAGAGTACATGCGACTGAGA-3′; reverse, 5′-GCTGTATCCTTCGCTGTTTCC’; TP53 forward, 5′-CAGCACATGACGGAGGTTGT-3′; reverse, 5′-TCATCCAAATACTCCACACGC-3′; CDKN1(p21^CIP1^)forward, 5′-TGTCCGTCAGAACCCATGC-3′; reverse, 5′-AAAGTCGAAGTTCCATCGCTC-3′; GAPDH forward, 5′- GGAGCGAGATCCCTCCAAAAT-3′; reverse, 5′-GGCTGTTGTCATACTTCTCATGG-3′. For knockdown experiments, cells were transiently transfected by siRNA pools with TransIT-X2 transfection reagent (Mirus, Madison, WI). Control-siRNAs were from Santa Cruz (sc-37007). EZH2-siRNAs were from Cell Signaling Technology (6509), CEBPB-siRNAs were from Santa Cruz (sc-29229) and SUZ12-siRNA (h) were from Santa Cruz (sc-45597).

### Western blotting

Cells were lysed with lysis buffer (100 mM Tris–HCl, pH 6.8, 100 mM DTT, 1% SDS, 10% glycerol). Proteins were separated by 10–12% SDS-PAGE, and transferred to PVDF membranes. Membranes were blocked in 5% non-fat milk in phosphate-buffered saline (PBS) for 1 h before incubation with primary antibody overnight at 4 °C. Membranes were washed and blocked with 5% milk and incubated with different primary antibodies overnight at 4 °C, followed by incubation with secondary antibodies. The primary antibodies used in western blotting included anti-Fos B(F-7) (sc-398595; Santa Cruz; 1:1000 dilution), anti-CEBPB (sc-7962; Santa Cruz; 1:1000 dilution), anti- histone H3K27me3 (ab6002, Abcam; 1:1000 dilution), anti-histone H3K27ac (#8173, Cell Signaling; 1:1000 dilution) and anti-GAPDH (sc-47724; Santa Cruz; 1:5000).

### Luciferase reporter and chromatin immunoprecipitation (ChIP)

The promoter region of FOSB gene was amplified from the genomic DNA of 293T cells and inserted into pGL4.15 vector (Promega, Madison, Wisconsin, USA). For the luciferase reporter assays, HEK293T cells were seeded in 24-well plates and transfected with the indicated plasmids using Lipo2000 for 36 h. Luciferase activity was measured using the Dual Luciferase Reporter Assay System (Promega). The firefly luciferase luminescence data were normalized by the Renilla luciferase luminescence data. A chromatin immunoprecipitation assay kit was used (Millipore, USA). In brief, cells fixed with 1% formaldehyde (Sigma, USA) and harvested in SDS lysis buffer. DNA was sheared to fragments of 200–1000 bp by sonications. Lysates containing soluble chromatin were incubated and precipitated overnight with 2 μg of anti- histone H3K27me3 (ab6002, Abcam), anti-histone H3K27ac (#8173, Cell Signaling), anti-EZH2 (E7031, Sigma-Aldrich); anti-C/EBPβ (sc-7962, Santa Cruz) or rabbit IgG (#ab172730, Abcam). Protein G agarose was then added for 4 h. Protein-DNA crosslinks were reversed by treatment with proteinase K for 2 h at 45 °C. The DNA was subsequently purified, diluted and subjected in the quantitative real-time PCR reactions.

### Construction of stable cell line

HEK293T cells were co-transfected with the vector control or pBabe-FOSB plasmids and packaging vectors for 48 h. Filtered viral supernatants were then collected and added to MDA-MB-436 cells with 10 μg/ml Ploybrane for 48 h and selected with puromycin (2 μg/ml) for 2 weeks.

### CRISPR/Cas9 knock out (KO) cell lines

The FOSB knock-out MDA-MB-231 cells were generated by CRISPR/Cas9 technology [27]. Briefly, single-stranded DNA oligonucleotides targeted to the coding sites of the gene was designed according to the Crisprgold website (https://crisprgold.mdc-berlin.de/index.php). Annealed primers were cloned into the vector PX459. Primers used for FOSB were list: Forward primer: CACCGTCGTAGGGGTCGACGACCGG). Reverse primer: AAACCCGGTCGTCGACCCCTACGAC). MDA-MB-231 cells were transfected with PX459-FOSB plasmid using Lipo2000 following the manufacturer’s instructions. Cells were selected with 2 µg/ml puromycin for about 2 weeks. Single clones were then selected and the knockout efficiency was verified by western blot assay with anti-FOSB antibody.

### Cell colony formation assay

Cells were counted, plated in triplicate at a density of 1000 per well in 6-well plates, and cultured in complete medium for about 2 weeks. Then, the cells were washed with PBS and fixed in methanol and stained with crystal violet. The numbers of colonies were then counted.

### Xenograft assays

Animal study was approved by the Animal Care and Use Committee of Liaoning Cancer Hospital and Institute. Four-week-old female BALB/cA nude mice were purchased from National Rodent Laboratory Animal Resources (Shanghai, China). All mice were kept in a specific pathogen-free facility. 1 × 10^7^ FOSB WT or KO MDA-MB-231 cells were suspended in 50 µl of DMEM medium, mixed 1:1 with matrigel and injected into the flanks of female nude mice. Tumor sizes were measured by a caliper and calculated using the formula length × width 2 × 1/2. Tumor weights were measured after mice were sacrificed.

### Statistical analyses

All experiments were at least repeated three times. Data are presented as mean ± standard deviation (SD). Results were analyzed using either two-tailed Student’s t test or two-way analysis of variance (ANOVA) in Graphpad Prism 7.0 software to assess statistical significance. P < 0.05 were considered statistically significant. Statistical significance is displayed as *P < 0.05, **P < 0.01, or ***P < 0.001.

## Results

### Genetic ablation or pharmacological inhibition of EZH2 resulted in FOSB gene expression in TNBC cells

**Fig. 1:**
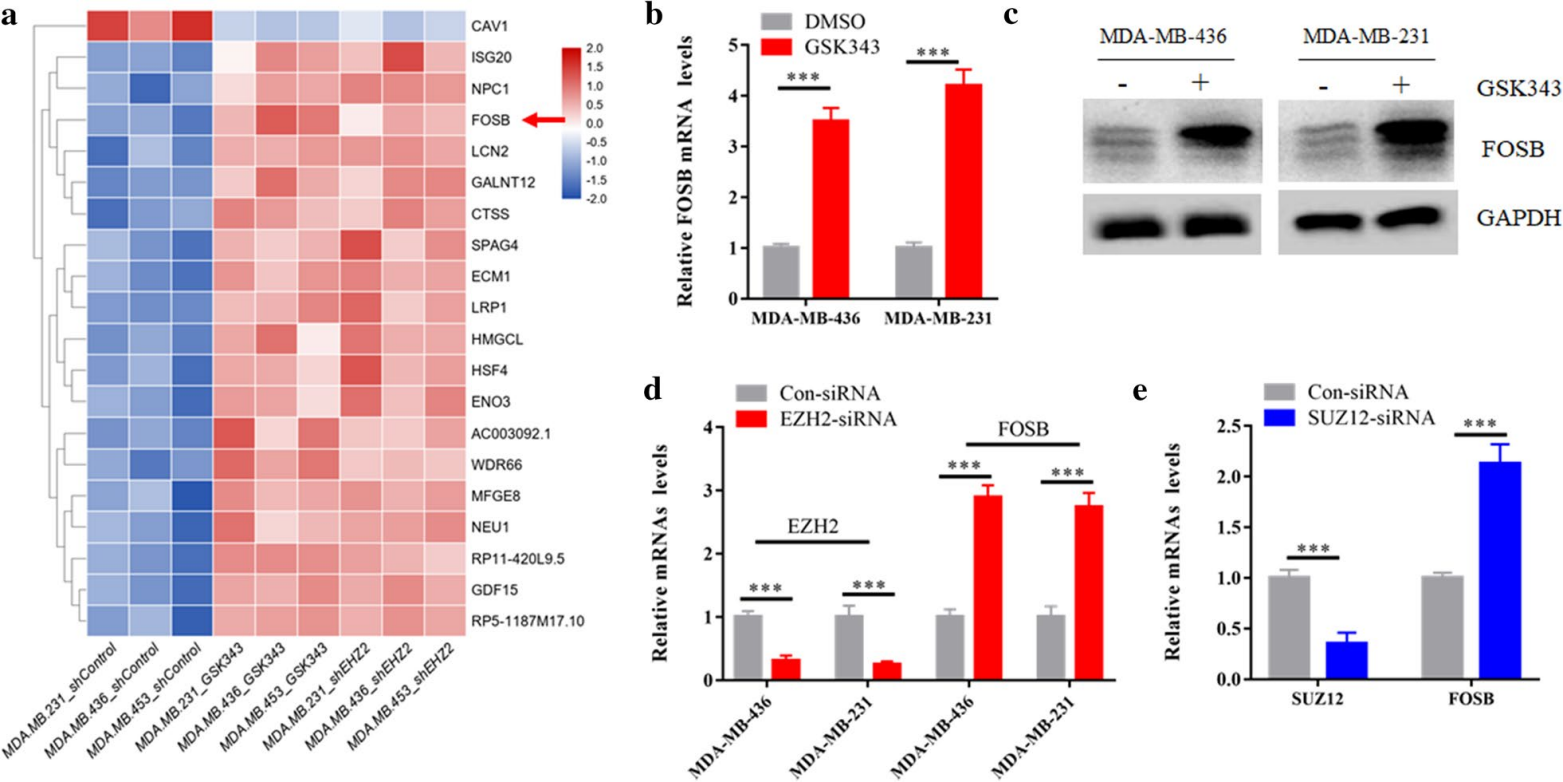
Genetic ablation or pharmacological inhibition of EZH2 resulted in FOSB gene expression in TNBC cells. **a** Heatmap demonstrated the common differentially expressed genes in MDA-MB-231, MDA-MB-436 and MDA-MB-453 cells either treated with 2 μM GSK343, or stably transduced with shEHZ2, compared to untreated controls. **b** The mRNAs of FOSB in MDA-MB-231 and MDA-MB-436 cells with or without 2 μM GSK343 treatment were determined by real-time PCR assay. **c** Immunoblot analysis of the indicated proteins in lysates from cells as in (**a**) with GAPDH as loading controls. **d** The mRNA levels of FOSB and EZH2 in MDA-MB-231 and MDA-MB-436 cells transfected with con-siRNA or siRNA against EZH2 were determined by real-time PCR assay. **e** The mRNAs of FOSB and SUZ12 in MDA-MB-231 cells transfected with con-siRNA or siRNA against SUZ12 were determined by real-time PCR assay

To identify novel targets of EZH2 in TNBC, we firstly reanalyzed the RNA sequencing analysis (RNA-seq) data of 3 TNBC cell lines either treated with 2 μM GSK343, or stably transduced with shEHZ2, compared with untreated controls (GSE112378) [28]. We assumed that genetic ablation or pharmacological inhibition of EZH2 might cause the increase the expression of EZH2 target genes. The Limma R package identified 20 common differentially expressed genes which were selected to draw a heat map (Fig. 1a). Among the up-regulated genes, we are particularly interested in FOSB, a member of the activator protein-1 complex. As FOSB has been reported to be activated in several kinds of tumors, we hypothesized that FOSB may be a critical downstream target gene of EZH2 and mediate the function of EZH2is in TNBC. Consistent with the RNA-seq data, we found that GSK343 treatment significantly increased FOSB mRNA and protein expression in MDA-MB-231 and MDA-MB-436, two TNBC cell lines (Fig. 1b, c). Similarly, knockdown of EZH2 in both cells by siRNAs also promoted FOSB mRNA expression (Fig. 1d). Moreover, knockdown of the SUZ12 subunit of the PRC2 complex by siRNAs also induced FOSB upregulation [29] (Fig. 1e), suggesting FOSB is regulated by PRC2 complex.

### FOSB is downregulated and associated with better relapse-free survival in TNBC

**Fig. 2:**
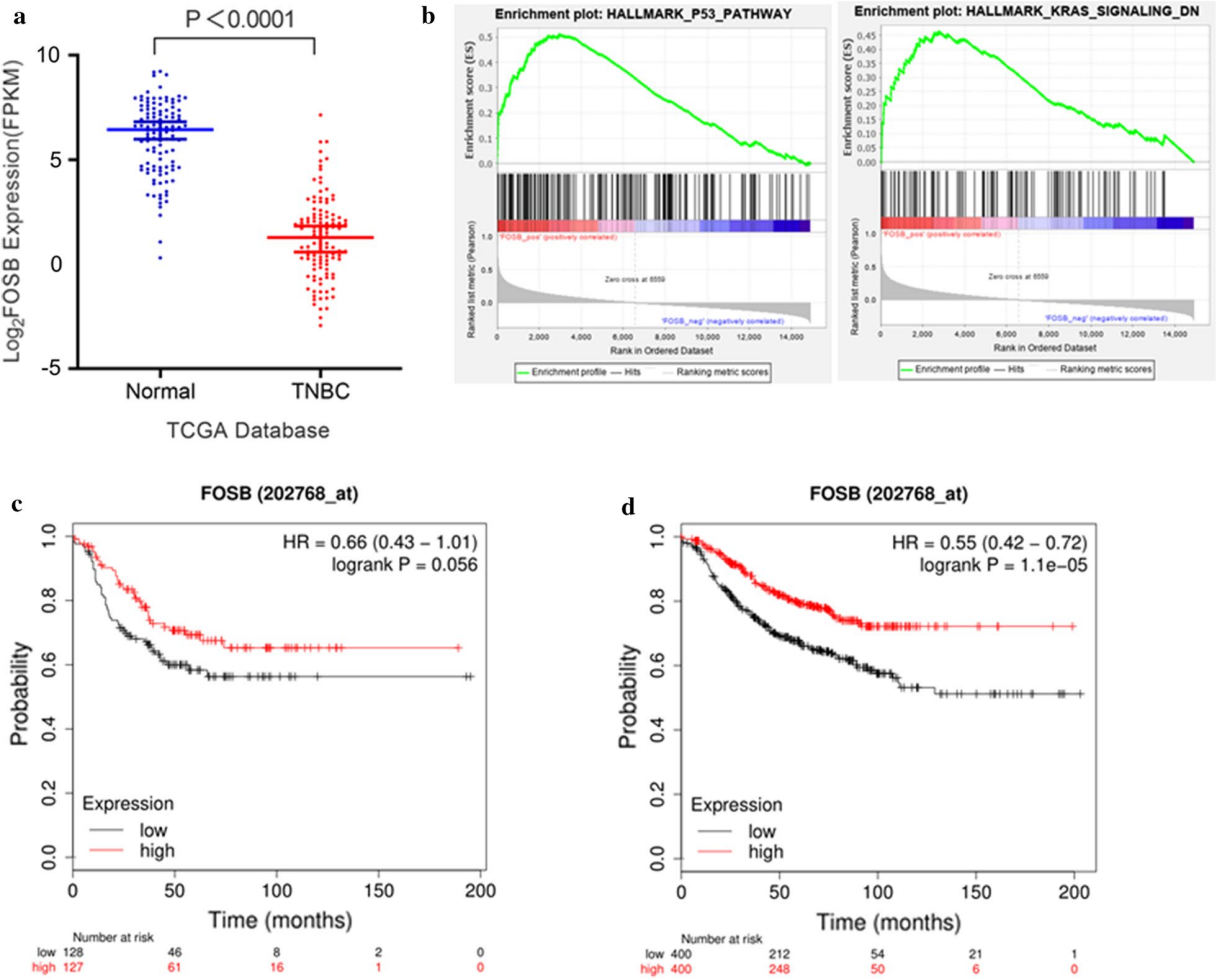
FOSB is downregulated and associated with better relapse-free survival in TNBC. **a** The mRNA expression of FOSB between 115 TNBC and 113 non‐tumor tissues in TCGA dataset. **b** GSEA plot of HALLMARK_P53 and KRAS_SIGNALING_down pathways enrichment in 115 TNBC samples with lower FOSB expression. **c** Survival curve of FOSB is plotted for TNBC patients. **d** Survival curve of FOSB is plotted for Her2-negative breast cancer patients

To understand the clinic relevance of FOSB, we firstly investigated its expression in TNBC. Analysis of Oncomine datasets revealed that FOSB was significantly downregulated in a variety of breast cancer samples (Additional file 1: Figure S1). Moreover, the downregulation of FOSB in TNBC was also confirmed in TCGA dataset (113 normal vs 115 TNBC, *P *< 0.0001) (Fig. 2a). By conducting gene set enrichment analysis (GSEA) of the RNA-seq data of those 115 TNBC, we found that HALLMARK_P53 and KRAS_SIGNALING_down pathways were significantly enriched, suggesting FOSB expression might associate with these tumor suppressing pathways (Fig. 2b). Next, we evaluated the prognosis value of FOSB in http://kmplot.com/analysis/. A survival analysis of the clinical breast cancer datasets indicated a strong correlation between higher FOSB expression and better relapse-free survival (RFS) (HR = 0.66, 95% CI 0.43–1.41, *P* = 0.056) (Fig. 2c) in TNBC. Importantly, in Her2-negative breast cancer, a significant correlation between higher FOSB expression and better overall-survival was also observed (HR = 0.55, 95% CI 0.42–0.72, *P* = 1.1e−05) (Fig. 2d). Together, these data suggested that aberrant expression of FOSB in TNBC is associated with better prognosis.

### H3K27me3 contributes to FOSB transcription silencing in TNBC

**Fig. 3:**
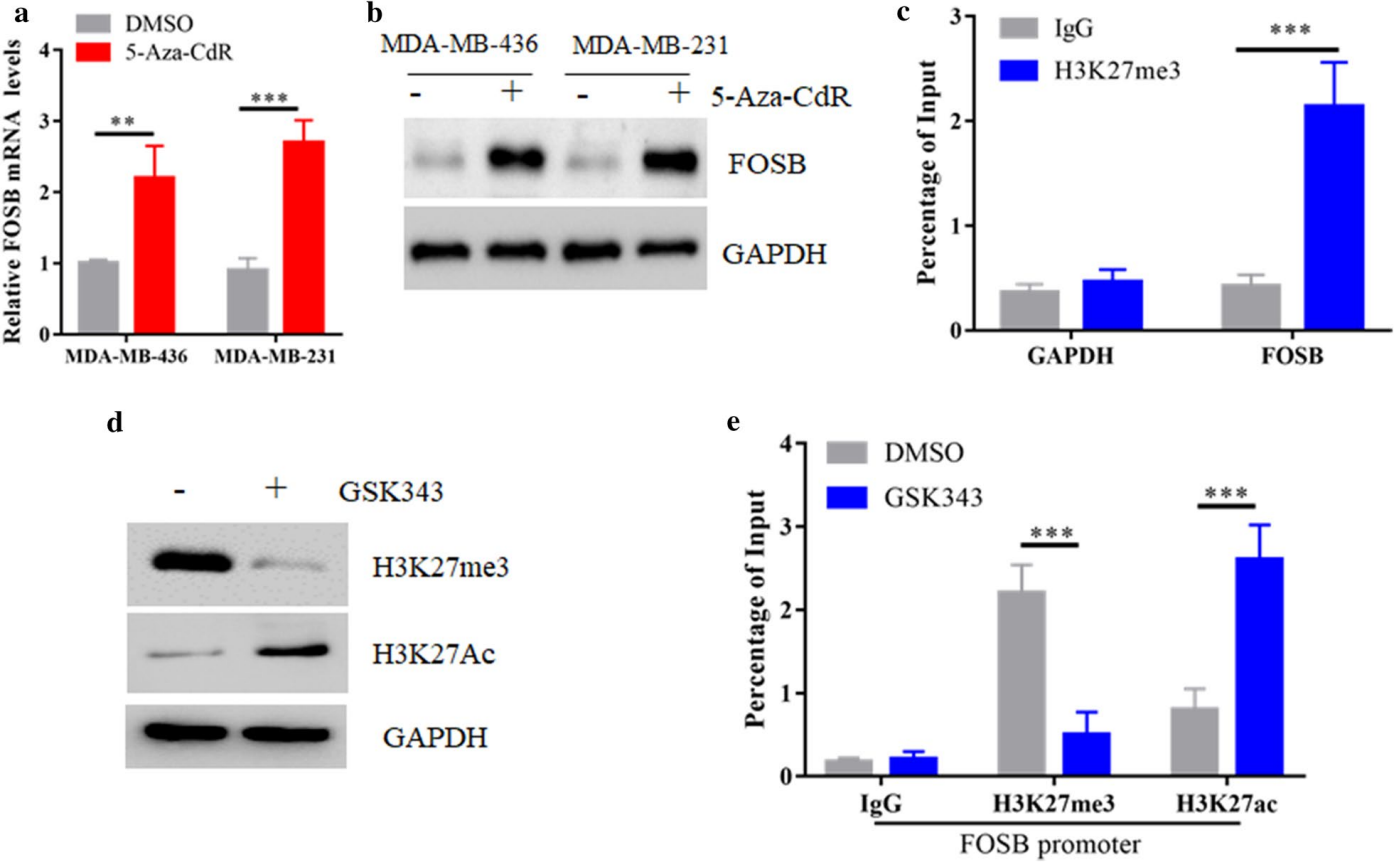
H3K27me3 contributes to FOSB transcription silencing in TNBC. **a** The mRNAs of FOSB in MDA-MB-231 and MDA-MB-436 cells with or without 10 μM 5-Aza-CdR treatment were determined by real-time PCR. **b** Immunoblot analysis of the protein levels of FOSB in (**a**). **c** ChIP shows enrichment of H3K27me3 at the FOSB promoter in MDA-MB-231 cells. **d** Immunoblot analysis of the H3K27me3 and H3K27Ac protein levels in MDA-MB-231 cells with or without 2 μM GSK343 treatment. **e** ChIP assay shows treatment with 2 μM GSK343 in MDA-MB-231 cells depleted H3K27me3 and increased H3K27ac from the FOSB promoter

EZH2-mediated H3K27me3 modification can further recruit methyltransferases to catalyze the methylation of CpG islands in the corresponding regions, so DNA methylation inhibitors could increase the expression of some PRC2 downstream genes expression [30]. To test this possibility, MDA-MB-231 and MDA-MB-436 cells were treated with a DNA methylation inhibitor 5-Aza-CdR (5-Aza-2′-deoxycytidine). As expected, 5-Aza-CdR treatment significantly promoted FOSB mRNA and protein expression in both TNBC cells (Fig. 3a, b). To further determine whether EZH2-mediated H3K27me3 plays a direct role in FOSB gene silencing, we examined whether the H3K27me3 mark is enriched at the FOSB promoter. Chromatin immunoprecipitation (ChIP) assays showed abundant promoter binding of H3K27me3 in MDA-MB-231 cells (Fig. 3c). Treatment with GSK343 could induce a global shift from trimethylation to acetylation at the H3K27 mark that to activate genes expression in the cells (Fig. 3d) [22]. Importantly, our ChIP assays confirmed the same changes at the FOSB promoter (Fig. 3e), which might contribute to the abundant reactivation of FOSB expression.

### C/EBPβ is required for FOSB gene epigenetic transcription activation

**Fig. 4:**
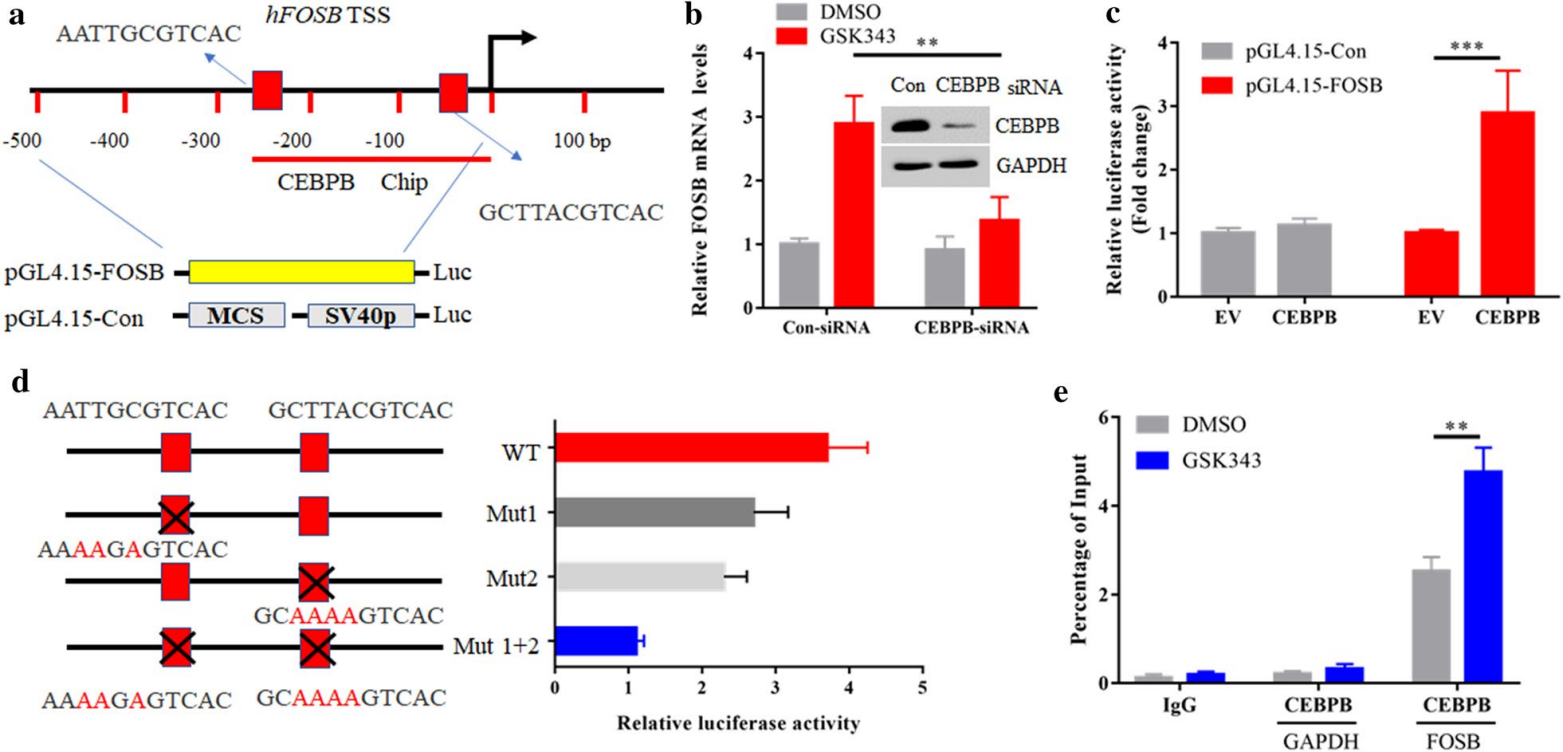
C/EBPβ is required for FOSB gene epigenetic transcription activation. **a** Schematic diagram shows FOSB gene promoter and putative C/EBPβ binding sites. *TSS* transcription start site. **b** MDA-MB-231 cells transfected with con-siRNA or siRNA against C/EBPβ were treated with or without 2 μM GSK343. The mRNA levels of FOSB in were determined by real-time PCR. The siRNA efficiency against C/EBPβ was determined by immunoblotting. **c** Overexpression of C/EBPβ activates FOSB promoter-driven luciferase activity. pGL4.15-Con or pGL4.15-FOSB plasmids were co-transfected with either empty vector (EV) or C/EBPβ in 293T cells. The luciferase activity was then measured. **d** The FOSB gene promoter contains two potential binding sites for C/EBPβ. Point mutations were highlighted with black cross, and the mutated residues were also highlighted in red. The transcriptional activity of wild-type or mutant FOSB promoters in 293T cells overexpressing C/EBPβ was then determined

We next investigated which transcriptional factors are involved in EZH2is-mediated FOSB gene reactivation. Analysis of the human FOSB promoter sequence (− 1000 to + 1 bp relative to TSS) for potential transcription factor binding sites by the eukaryotic promoter database (EPD) predicted two binding sites for C/EBPβ (Fig. 4a). C/EBPβ can bind and recruit histone acetyltransferases p300 and CBP, which could catalyze H3K27 acetylation (H3K27ac) [31]. To test whether C/EBPβ is involved in FOSB reactivation, the expression of C/EBPβ was silenced by siRNAs and then treated GSK343. We found that knockdown of C/EBPβ significantly blocked GSK343-mediated FOSB expression (Fig. 4b). To further test whether C/EBPβ can directly activate FOSB transcription, we co-transfected C/EBPβ with a luciferase reporter vector under the control of the FOSB promoter (− 500 to + 1 bp of TSS) in 293T cells. C/EBPβ could induce about threefold activation of luciferase activity (Fig. 4c), confirming that C/EBPβ can directly activate FOSB transcription. As two potential binding sites were existed in FOSB promoter region, we then determined which one is essential for FOSB expression. We found that mutation of either C/EBPβ binding site reduced, while mutation of both sites largely blocked the activity of FOSB promoter by C/EBPβ (Fig. 4d). Moreover, ChIP assays revealed that GSK343 treatment could promote abundant binding of C/EBPβ to the endogenous FOSB promoter (Fig. 4e). Taken together, these results indicate that epigenetic reactivation of FOSB expression by EZH2 inhibition mainly through two functional C/EBPβ binding sites in TNBC.

### FOSB exhibits tumor-suppressor functions in TNBC cells

**Fig. 5:**
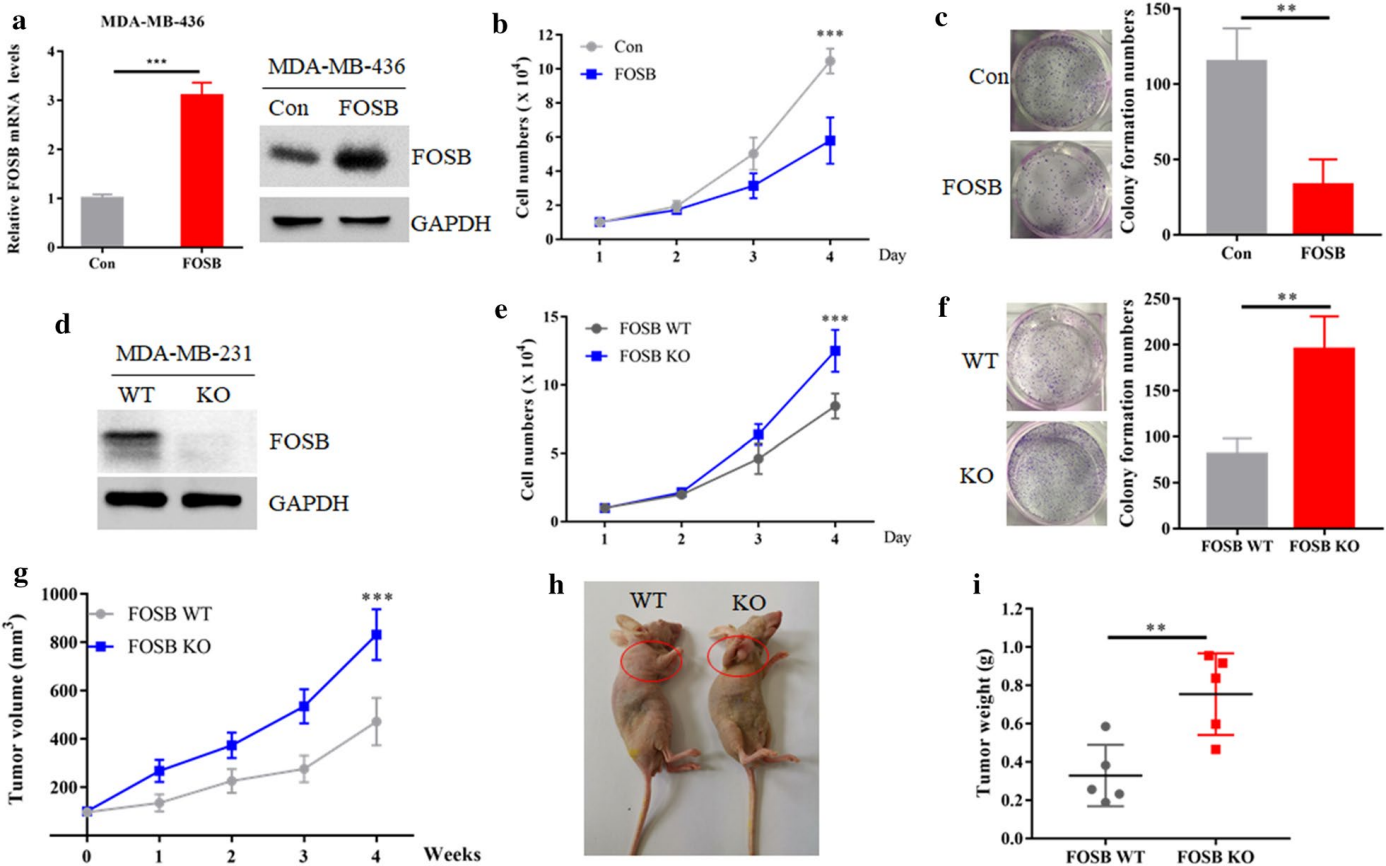
FOSB exhibits tumor-suppressor functions in TNBC cells. **a** The mRNA and protein levels of FOSB from MDA-MB-231 cells stable expression of pcDNA3(Con) or pcDNA3-FOSB were detected by real-time PCR assay and western blot, respectively. **b** The cell growth curve of MDA-MB-231 cells stable expression of pcDNA3(Con) or pcDNA3-FOSB. **c** Clonogenic assay of MDA-MB-231 cells stable expression of pcDNA3(Con) or pcDNA3-FOSB. 1000 cells were seeded into each well of a six-well plate, and cultured for about 2 weeks. Cells were stained with crystal violet and then counted. **d** FOSB KO MDA-MB-231 cells were generated by CRISPR assay and detected by western blot. **e** The cell growth curve of FOSB WT and KO MDA-MB-231 cells. **f** Clonogenic assay of FOSB WT and KO MDA-MB-231 cells. 1000 cells were seeded into each well of a six-well plate, and cultured for about 2 weeks. Cells were stained with crystal violet and then counted. **g** Each nude mouse was subcutaneously injected with 1 × 10^7^ FOSB WT and KO MDA-MB-231 cells for about 4 weeks. Tumour growth was measured using a caliper at the indicated times after injection. n = 5 for each group. ***P < 0.001. **h** The image shows representative tumor-bearing mice for each group. **i** Mice tumor weights in each group were measured after mice were sacrificed. **P < 0.01

The GSEA results suggest FOSB might have a role in TNBC cells growth control (Fig. 2b). To this end, we firstly generated a FOSB overexpressing MDA-MB-436 cell line. The mRNA expression level of exogenous FOSB is about 3.5 times, which is close to the endogenous expression level in MDA-MB-436 cells induced by GSK343 (Fig. 5a). Overexpression of FOSB caused significantly decreased cell growth and colony formation ability (Fig. 5b, c). We further constructed a FOSB knock out (KO) MDA-MB-231 cell line using CRISPR–Cas9 technology to completely deplete FOSB gene (Fig. 5d), and found that FOSB KO cells showed increased proliferation and colony formation ability when compared with the control wild type (WT) cells (Fig. 5e, f). We next expanded our study to xenograft models to investigate whether FOSB affected TNBC cells proliferation in vivo. BALB/c nude mice were subcutaneously injected with 1 × 10^7^ FOSB WT or KO MDA-MB-231 cells for up to 4 weeks. Nude mice experiments confirmed that knock out of FOSB markedly increased MDA-MB-231 cells tumor growth (Fig. 5g–i). Thus, these data indicated that FOSB played a tumor-suppressor role in the regulation of TNBC cells proliferation both in vitro and in vivo.

### EZH2is reduce TNBC cell growth in a FOSB-dependent fashion

**Fig. 6:**
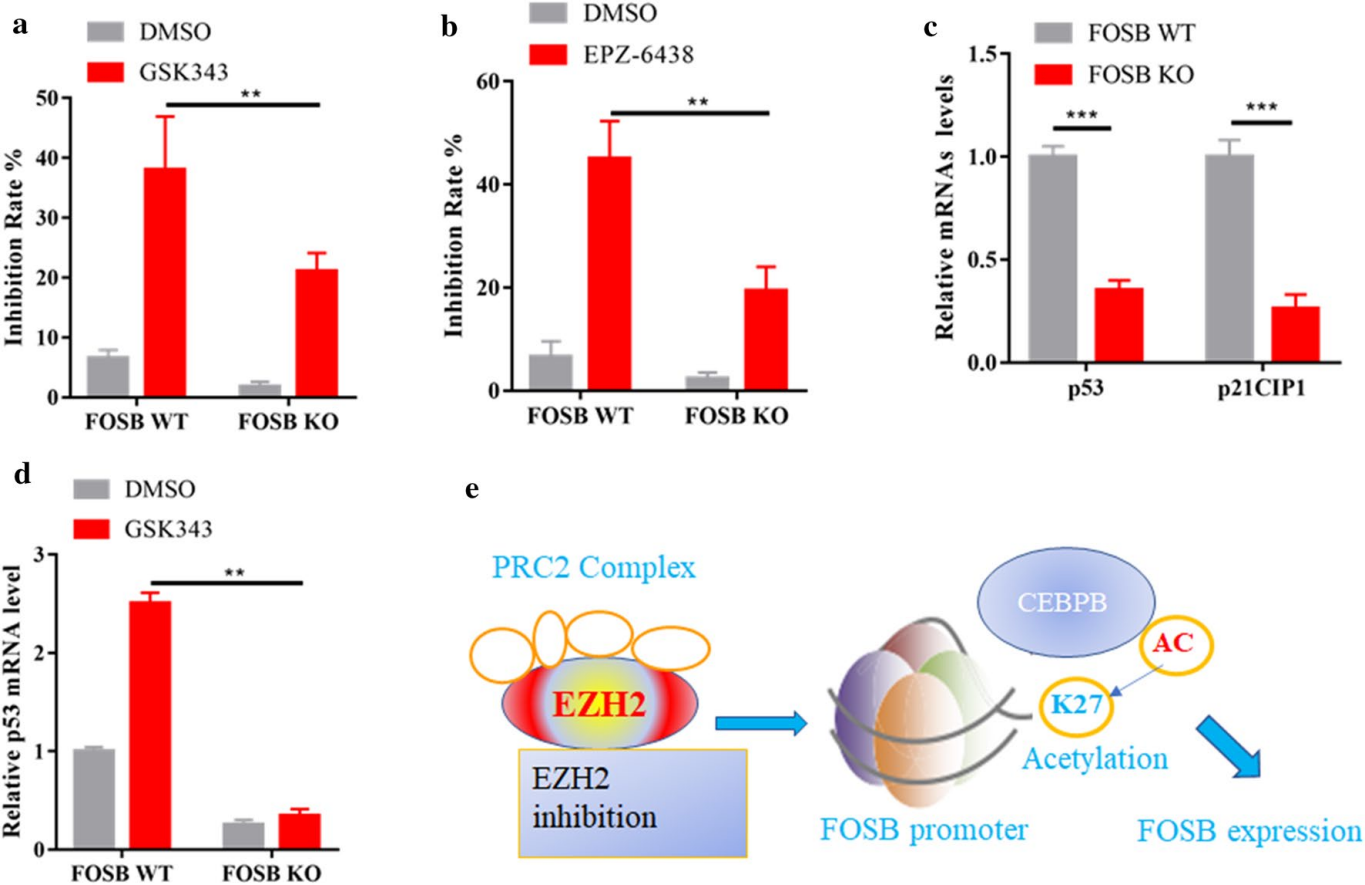
EZH2is reduce TNBC cell growth in a FOSB-dependent fashion. **a** Cell growth assay. FOSB WT and KO MDA-MB-231 cells were exposed to 2 μM GSK343 for 6 days. Cell growth inhibition rate was measured by CCK8 assay. n = 3 replicates. **b** FOSB WT and KO MDA-MB-231 cells were exposed to 5 μM EPZ-6438 for 6 days. Cell growth inhibition rate was measured by CCK8 assay. n = 3 replicates. **c** The mRNA levels of p53 and p21^CIP1^ in FOSB WT and KO MDA-MB-231 cells were determined by real-time PCR assay. **d** FOSB WT and KO MDA-MB-231 cells were treated with or without 2 μM GSK343. The mRNA levels of p53 in determined by real-time PCR assay. **e** Proposed model for the action of EZH2is. EZH2is promoted the shift from H3K27me3 to H3K27ac at the FOSB promoter, and recruited the transcription factor C/EBPβ to activate FOSB gene transcription

It has been reported that lower concentrations of GSK343 (0.5 μM and 2 μM) consistently inhibited the proliferation of TNBC cell lines [28]. To test whether FOSB affected the sensitivity of TNBC cells to GSK343 treatment, we treated FOSB WT and KO MDA-MB-231 cells with 2 μM GSK343. We found that knock out of FOSB significantly abrogated the antiproliferative effect of GSK343 (Fig. 6a). The similar results were also observed by using an another EZH2 inhibitor EPZ-6438 (Fig. 6b), suggesting that EZH2is-depended TNBC cell growth retardant required FOSB reactivation. It has been reported that depletion of p53 significantly affected the anti-proliferation role of EZH2is in TNBC cells [28]. We found that the mRNA levels of p53 and its downstream target CDKN1A (p21^CIP1^) were significantly decreased in FOSB KO MDA-MB-231 cells (Fig. 6c). Moreover, GSK343 treatment failed to induced the expression of p53 in FOSB KO cells (Fig. 6d). Together, these data indicated that FOSB-mediated p53 expression is required for the antiproliferative effect of EZH2is in TNBC cells.

## Discussion

In the present study, we report that EZH2-mediated epigenetic inactivation of FOSB in TNBC cells and FOSB expression is correlated with better relapse-free survival in TNBC tissues. In line with these observations, we showed that genetic inhibition of FOSB promoted TNBC cells proliferation in vitro, and thus tumor progression in subcutaneously transplanted TNBC mice, indicating a critical role of FOSB in the regulation of TNBC progress. After EZH2is treatment, FOSB expression was significantly increased, a process required the involvement of the transcriptional factor C/EBPβ. Indeed, two potential C/EBPβ binding sites were located in the promoter region of FOSB gene. Thus, our data clearly showed that upon EZH2is treatment, the transition from H3K27me3 to H3K27ac at the promoter of FOSB gene recruited C/EBPβ to facilitate FOSB gene expression in TNBC. Importantly, we found that FOSB-depleted TNBC cells were resistant to EZH2is treatment, implying loss of FOSB might confer drug resistance to EZH2is. Thus, our results suggested that EZH2-mediated epigenetic inactivation of FOSB promoted TNBC expression (Fig. 6e), demonstrating that reactivation of FOSB expression underlies the anti-TNBC action of EZH2is.

There are still some issues to be clarified. For instance, the sample size in our present study is relatively small. Further studies using larger clinic TNBC samples are still needed to confirm the relationship between EZH2 and FOSB. Moreover, it might be interested and important to study the relationship between EZH2 and other AP-1 family members in the future.

In addition to its known roles in histone modification and transcriptional regulation, EZH2 has been reported to methylate non-histone substrates. EZH2 binds and methylates STAT3 to promote tumorigenicity of glioblastoma [32]. EZH2 can bind and methylate androgen receptor (AR) to modulate AR recruitment to the sites bound by both AR and EZH2 [33]. EZH2 could also promote monomethylation of RORa to mark it for degradation by the DCAF1/DDB1/CUL4 E3 ubiquitin ligase complex [34]. Although we found that EZH2is could promote the shift from H3K27me3 to H3K27ac at FOSB promoter to facilitate FOSB gene expression, we could not exclude the possibility that EZH2 might also bind and methylate other non-histone substrate to regulate FOSB expression.

## Conclusion

Together, our in vivo and in vitro results demonstrate that targeting of EZH2 in TNBC might cause epigenetic reactivation of FOSB genes and activation of p53 pathway, providing a novel mechanism of EZH2is treatment in TNBC. Our work shows that FOSB might be the major effector of EZH2is in TNBC, and that the therapeutic effects of EZH2is will be lost in the absence of FOSB. Thus, we can predict that the FOSB negative TNBC will not respond to EZH2is treatment and lead to therapeutic resistance. The future studies will need to include strategies to counter emergence of EZH2is resistance in FOSB negative TNBC patients.

## Supplementary information

**Additional file 1: Figure S1.** FOSB was significantly downregulated in a variety of breast cancer samples in Oncomine datasets.

## Data Availability

The GEO data we used is GSE112378.

## Abbreviations

H3K27me3: Histone 3 trimethylated on lysine 27
EZH2i: EZH2 inhibitor
RFS: Relapse-free survival
TNBC: Triple-negative breast cancer
Chip: Chromatin immunoprecipitation
ER: Estrogen receptor
PR: Progesterone receptor
HER2: The human epidermal growth factor receptor 2
GCB: Germinal center B cell
DLBCL: Diffuse large cell B-cell lymphomas
FL: Follicular lymphomas
