# Supplementary material for: EZH2 inhibitors-mediated epigenetic reactivation of FOSB inhibits triple-negative breast cancer progress
Source: Cancer Cell Int. 2020 May 19;20:175. doi: 10.1186/s12935-020-01260-5 (PMC7236314; doi:10.1186/s12935-020-01260-5)
Supplement: Supplementary file 1 — Additional file 1: Figure S1. FOSB was significantly downregulated in a variety of breast cancer samples in Oncomine datasets. [file 12935_2020_1260_MOESM1_ESM.docx]

Additional Figure legends

Additional Figure S1 FOSB was significantly downregulated in a variety of breast cancer samples in Oncomine datasets


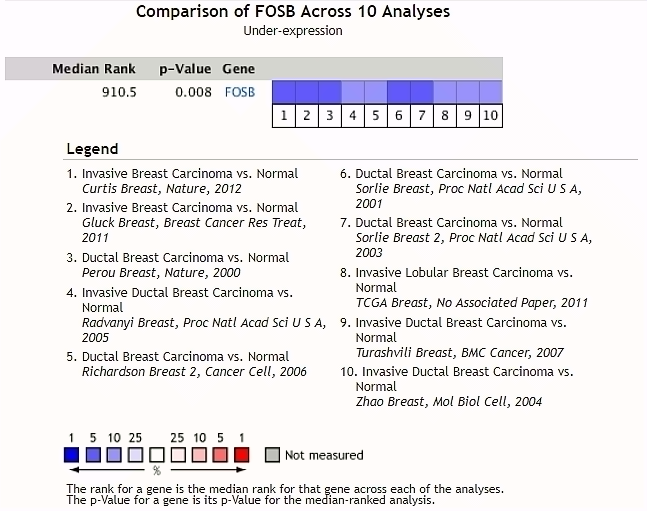


We searched for breast cancer related research in the Oncomine database by clicking FOSB gene and we found that FOSB was significantly lowered in 10 breast cancer databases, which are shown in the legend.
